# Supplementary figures and images for: α7 nicotinic acetylcholine receptor interaction with G proteins in breast cancer cell proliferation, motility, and calcium signaling
Source: PLoS One. 2023 Jul 25;18(7):e0289098. doi: 10.1371/journal.pone.0289098 (PMC10368273; doi:10.1371/journal.pone.0289098)

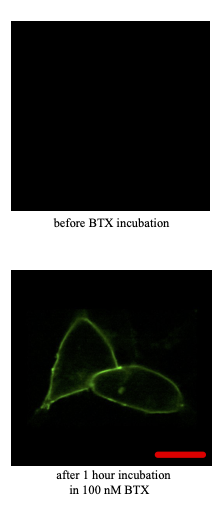

Supplement: S1 Fig — MCF-7 cells were labeled with 100 nM BTX. Cell membranes were not permeabilized in these labeling experiments. Bottom panel shows a representative image of a labeled cell. Scale bar = 5μm. (TIFF) [file pone.0289098.s001.tiff]
